# Supplementary material for: Use, Abuse, and Misuse of Nasal Medications: Real-Life Survey on Community Pharmacist’s Perceptions
Source: J Pers Med. 2023 Mar 26;13(4):579. doi: 10.3390/jpm13040579 (PMC10142332; doi:10.3390/jpm13040579)
Supplement: Supplementary file 1 [file jpm-13-00579-s001.zip › jpm-2273560-supplementary.pdf]

Supplement index

Table S1: Distribution over the Italian territory of the included pharmacies.

	n	%
Nord	152	40.4
Nord-est	55	14.6
Veneto	27	7.2
Friuli-Venezia Giulia	10	2.7
Emilia-Romagna	9	2.4
Trentino-Alto Adige	9	2.4
Nord-ovest	97	25.8
Piemonte	18	4.8
Valle D'Aosta	1	0.3
Liguria	16	4.3
Lombardia	62	16,5
Center	81	21.5
Marche	8	2.1
Toscana	6	1.6
Umbria	1	0.3
Lazio	66	17.6
Sud	143	38.1
Abruzzo	15	4.0
Basilicata	3	0.8
Molise	5	1.3
Campania	21	5.6
Puglia	22	5.9
Calabria	24	6.4
Islands	53	14.1
Sardegna	37	9.8
Sicilia	16	4.3
Overall	376	100
